# Oxidative stress induced by berberine-based mitochondria-targeted low temperature photothermal therapy

**DOI:** 10.3389/fchem.2023.1114434

**Published:** 2023-02-02

**Authors:** Hongzhi Hu, Qingcheng Song, Wenbo Yang, Qianwen Zeng, Zihui Liang, Weijian Liu, Zengwu Shao, Yiran Zhang, Chao Chen, Baichuan Wang

**Affiliations:** ^1^ Department of Orthopaedics, Union Hospital, Tongji Medical College, Huazhong University of Science and Technology, Wuhan, China; ^2^ Department of Orthopaedic Surgery, The Third Hospital of Hebei Medical University, Shijazhuang, China; ^3^ School of Nursing, Tongji Medical College, Huazhong University of Science and Technology, Wuhan, China; ^4^ Ministry of Education Key Laboratory for the Green Preparation and Application of Functional Materials, Faculty of Materials Science and Engineering, Hubei University, Wuhan, China; ^5^ School of Medicine, Nankai University, Tianjin, China

**Keywords:** oxidative stress, reactive oxygen species, berberine derivative, low temperature, mitochondria targeted, photothermal therapy

## Abstract

**Introduction:** Mitochondria-targeted low-temperature photothermal therapy (LPTT) is a promising strategy that could maximize anticancer effects and overcome tumor thermal resistance. However, the successful synthesis of mitochondria-targeted nanodrug delivery system for LPTT still faces diverse challenges, such as laborious preparations processes, low drug-loading, and significant systemic toxicity from the carriers.

**Methods:** In this study, we used the tumor-targeting folic acid (FA) and mitochondria-targeting berberine (BBR) derivatives (BD) co-modified polyethylene glycol (PEG)-decorated graphene oxide (GO) to synthesize a novel mitochondria-targeting nanocomposite (GO-PEG-FA/BD), which can effectively accumulate in mitochondria of the osteosarcoma (OS) cells and achieve enhanced mitochondria-targeted LPTT effects with minimal cell toxicity. The mitochondria-targeted LPTT effects were validated both *in vitro* and *vivo*.

**Results:** *In vitro* experiments, the nanocomposites (GO-PEG-FA/BD) could eliminate membrane potential (ΔΨm), deprive the ATP of cancer cells, and increase the levels of reactive oxygen species (ROS), which ultimately induce oxidative stress damage. Furthermore, *in vivo* results showed that the enhanced mitochondria-targeted LPTT could exert an excellent anti-cancer effect with minimal toxicity.

**Discussion:** Taken together, this study provides a practicable strategy to develop an ingenious nanoplatform for cancer synergetic therapy *via* mitochondria-targeted LPTT, which hold enormous potential for future clinical translation.

## 1 Introduction

Osteosarcoma (OS) is the most common primary malignant bone tumor occurring predominantly in children and adolescents (Hameed and Mandelker, 2018). With the development of modern imaging technologies, surgical techniques and chemotherapy, the 5-year overall survival rate of OS patients has increased to 78.6%. However, patients still face a high rate of recurrence and metastasis because of the continuously decreased therapeutic efficacy of chemotherapeutic drugs (Lu et al., 2018; Chen et al., 2021; Arafa et al., 2022). Moreover, the non-selective nature of chemotherapeutic drugs inevitably causes the uncontrolled side effects to normal tissues (Arafa et al., 2022). Thus, it is urgently needed to develop a promising therapeutic strategy with maximal therapeutic activity concomitantly with minimal toxic side effects for OS patients.

Precise delivery of anti-cancer drugs based on nano-drug delivery systems to subcellular targets, such as mitochondria, lysosomes, and nuclei, has already been considered as an excellent cancer treatment strategy (Yang L. et al., 2022; Zhang J. et al., 2022; Wang et al., 2022). Mitochondria, the powerhouse of aerobic metabolism, exert an essential role in cellular physiological and pathological processes (Song et al., 2019; Zhang S. et al., 2022). In the past decades, the central role of mitochondria in tumor progression and metastasis has been studied extensively. Of note, increasing evidences showed the strategy of delivering nano-anticancer drugs into mitochondria has emerged as a promising approach for cancer therapy (Song et al., 2019; Tan et al., 2022). However, the successful synthesis of mitochondria-targeting nano-drug delivery system still faces diverse challenges, such as laborious preparations processes (Shi et al., 2010), low drug-loading (Luo et al., 2016), and significant systemic toxicity from the carriers (Zhou et al., 2013; Lin et al., 2021). Therefore, the development of mitochondria-targeted multi-functional nano-drug delivery systems with facile synthesis, high drug envelopment rates, and good biocompatibility is imperative.

Berberine (BBR), a nature alkaloid isolated from berberis species, has been widely studied for its broad-spectrum anticancer activity (Li J. et al., 2022; Jin et al., 2022; Okuno et al., 2022). Accumulating evidence has also revealed that BBR exerted an excellent anti-OS effect with very low toxicity, indicating that it might be a potential anticancer drug candidate for OS (Yu et al., 2014; Chen, 2016; Mishra et al., 2020; Wang et al., 2020). Encouragingly, previous researches demonstrated that 9-O-octadecyl substituted BBR derivative (BD) exhibited outstanding mitochondria-targeting property in cancer cells (Tuo et al., 2016; Song et al., 2019; Lin et al., 2021). Accordingly, BD could be applied not only as a promising anti-cancer drug but also as an effective mitochondrial targeting ligand. However, it should be noticed that BBR possesses the disadvantages of poor aqueous solubility and low cell targeting ability, which currently hamper its clinical application (Majidzadeh et al., 2020). In addition, single-agent treatment might not be able to achieve satisfactory therapeutic effects due to the enhanced self-repair capability and increased drug efflux of tumor cells (Hu et al., 2010; Majidzadeh et al., 2020). Therefore, designing of a mitochondrial-targeted drug delivery system that simultaneously integrates other therapeutic modalities will be of great interest.

Phototherapy (PTT), a spatiotemporally controllable strategy, has emerged as a promising cancer therapeutic alternative therapeutic modality for cancers due to its unique advantages, including minimal invasiveness, high efficiency, and low systemic toxicity (Hu et al., 2021; Li S. et al., 2022; Mishra et al., 2022; Yang et al., 2023). It is well known that PTT utilizing near-infrared (NIR) laser irradiation converts light energy into heat energy through photothermal agents, which locally generates high temperatures to induce thermal ablation of tumor cells (Fan et al., 2022; Tunçel and Yurt, 2022; Zhong et al., 2022). Nevertheless, the thermal ablation during PTT might cause unwanted damages to the surrounding healthy tissues (Yang S. et al., 2022). Recently, low-temperature PTT (LPTT) has been proposed to circumvent the above mentioned limitation at temperatures that are safe for the human body (Ma et al., 2022). However, single LPTT usually fails to achieve satisfactory therapeutic effects, which might be ascribed to the heterogenous distribution of PTT agents in cancer cells, the limited penetration depth of light and the acquisition of thermoresistance in tumors (Hu et al., 2022; Ma et al., 2022). With all these findings in mind, we hypothesized that BD-mediated mitochondria-targeting LPTT would be a path-breaking strategy to acquire synergistic effects, overcome respective shortcomings, and decrease the therapeutic threshold for both the drug dosage as well as diminish damage to the surrounding normal tissues. Some studies including our previous ones have demonstrated that graphene oxide (GO) is a promising drug delivery system due to their large surface area, high ratio of drug loading, low cost, and logical biocompatibility (Deng et al., 2020; Huang et al., 2020). Moreover, GO has also proven to act as a photothermal agent, which converts the light energy into heat for cancer PTT (Dash et al., 2022). These promising properties make it possible to delivery BD into the mitochondria of the tumor and achieve a synergistic antitumor effect.

**SCHEME 1:**
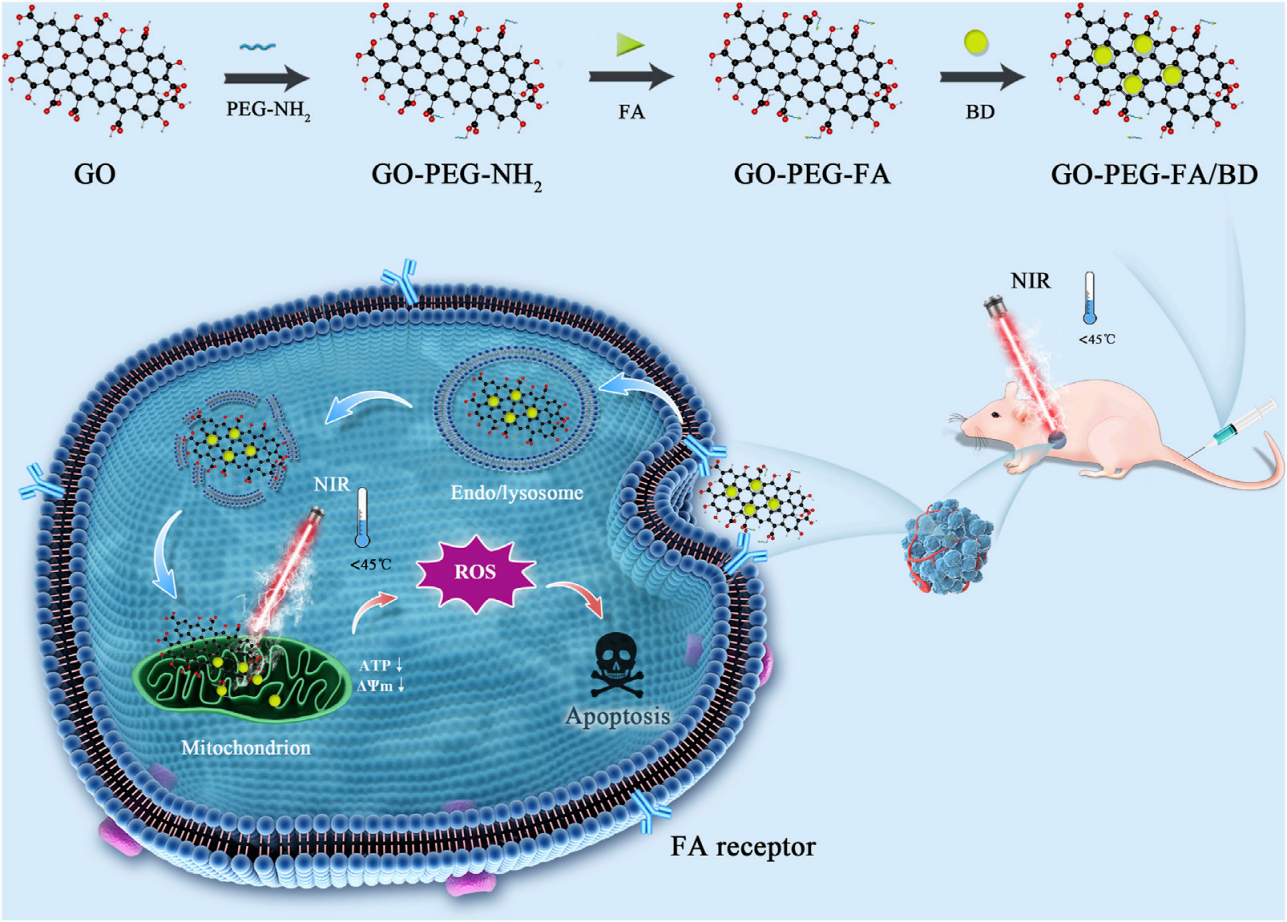
Schematic illustration of the preparation and application of GO-PEG-FA/BD nanocomposite.

Herein, we are committed to designing a mitochondria-targeting nano-platform based on a simple and facile preparation, which can achieve synergy of LPTT and anti-cancer drugs for highly potent cancer treatment. As shown in Scheme 1, we first developed poly (ethylene glycol) (PEG)-ylated GO and then modified with folic acid (FA) that binds specifically to folate receptor. It is widely known that the folate receptor is highly expressed in various tumor types, including OS (Ai et al., 2017; Meshkini and Oveisi, 2017; Hu et al., 2021). Subsequently, BD was loaded onto the surface of the tumor-targeted GO (GO-PEG-FA) *via* π−π stacking and hydrophobic interactions to obtain the final nanocomposite (GO-PEG-FA/BD). Once reaching the tumor site, GO-PEG-FA/BD, in theory, could ultimately target the mitochondria of cancer cells due to the active targeting capability of FA and the mitochondria-targeting ability of BD. In the present study, our results showed that GO-PEG-FA/BD was enriched in the tumor region, and effectively activated the intrinsic mitochondrial apoptosis pathways with a low temperature laser irradiation (808 nm). Taken together, this study provides a practicable strategy for cancer synergetic therapy *via* mitochondria-targeted LPTT.

## 2 Experimental section

### 2.1 Materials

N-hydroxysuccinimide (NHS), N-(3-dimethylaminopropyl)-N-ethylcarbodiimide hydrochloride (EDC), and folic acid (FA) were obtained from Sigma-Aldrich (St. Louis, MO, United States). BBR (purity >98%) was purchased from Solarbio (Beijing, China) and dissolved with dimethylsulfoxide (DMSO) (Sigma). Other chemicals were commercially available and used without further purification.

### 2.2 Preparation and characterization of GO-PEG-FA/BD

The design and synthesis of GO-PEG-FA/BD were illustrated in Scheme 1. Firstly, GO was synthesized by a modified Hummers method described elsewhere (Park and Ruoff, 2009; Huang et al., 2011). Subsequently, the GO-PEG-NH_2_ was prepared by an acetylation reaction between carboxyl groups in GO and amine groups in PEG-NH_2_ (Kim et al., 2016). And then, GO-PEG-NH_2_ was conjugated with FA according to the literature with mild modification (Huang et al., 2020). Briefly, EDC (100 mg) and NHS (50 mg) were dissolved in the DMSO solution of FA (2 mg/mL, 2 mL) and stirred for 2 h to activate the carboxyl groups of FA. Subsequently, the mixed solution was added into the DMSO solution of GO-PEG-NH_2_ (2 mg mL^−1^, 2 mL) and stirred overnight at room temperature, followed by dialysis against DI-water to remove the unreacted reactants. The product (GO-PEG-FA) was washed three times with DI-water and then freeze dried.

For BD loading, the BD was firstly prepared according to the method described in the previous study (Fu et al., 2015). After that, 10 mg GO-PEG-FA were first dispersed in BD (2 mg/mL in DMSO, 10 mL) by ultrasonication and then gently stirred at room temperature for 24 h. Subsequently, the BD-loaded GO-PEG-FA (GO-PEG-FA/BD) was collected by centrifugation at 12,000 rpm for 60 min and washed 3 times with DMSO to remove the unbound drug. The yielded NPs were then washed with PBS for 3 times and stored at 4°C before further use.

Scanning electron microscope (SEM) images were obtained with a transmission electron microscope (ZEISS Gemini 300). The surface charge of samples was evaluated by using a Zetasizer Nano ZS90 equipment (Malvern Instruments, United Kingdom).

### 2.3 Hemolysis assay

The hemolysis experiments were performed by detecting the released hemoglobin. In brief, 2.0 mL blood samples of healthy mice were extracted and anticoagulated. The red blood cells (RBCs) were firstly obtained after 5 min of centrifugation at 8,000 rpm under 4°C and 3 times of rinses with PBS and then diluted by adding 4.0 mL PBS. Afterwards, 0.8 mL PBS with various amounts of NPs was added into 0.2 mL the above RBCs suspension with the final concentration of NPs was 50, 100, 200, 400 and 800 μg mL^−1^, respectively. RBC suspension plus PBS or pure water without NPs were used as negative and positive control, respectively. After 2 h of incubation at 37°C under shaking, the mixed systems were centrifuged at 8,000 rpm and the absorbance at 541 nm of the obtained supernatant was determined with a UV-vis spectrophotometer. The hemolysis percentage of RBCs was calculated according to the protocol described elsewhere.

### 2.4 Cell culture

The MNNG/HOS cells (human OS cells) used in the experiment were purchased from Cell Bank of Shanghai Institute of Biochemistry. For incubation of the MNNG/HOS cells, α-modified essential medium (MEM) with 1% antibiotics (penicillin and streptomycin) and 10% fetal bovine serum (FBS) was used. The bone marrow stromal cells (BMSCs) were kindly provided by Dr. Song Gong (Tongji Medical College, Huazhong University of Science and Technology). The BMSCs were cultured with Dulbecco’s MEM (DMEM)/F12 containing 15% FBS and 1% penicillin–streptomycin. All the cells were maintained in a humidified incubator with 5% CO_2_ at 37°C.

### 2.5 Cell uptake study

To assess cancer cell uptake ability of the nanoparticles, cells were planted in a 6-well plate (1 × 10^5^ cells/well) and cultured at 37°C for 24 h. After 4 h of incubation with various samples (BD, GO-PEG/BD and GO-PEG-FA/BD) at equivalent concentration of BD (10 μg mL^−1^), the cells were incubated with Hoechst 33342 (10 μg mL^−1^) for 10 min and rinsed for three times with PBS. Subsequently, the fluorescence of cells was recorded by a fluorescence microscope.

### 2.6 Mitochondria targeting property

In brief, the MNNG/HOS cells were firstly seeded (1 × 10^5^/well) in six-well cell culture plates and incubated overnight. Subsequently, the cells were incubated with various samples for 6 h and then stained with Mito-Tracker Red (Beyotime Biotechnology, China) as well as Hoechst 33432 (10 μg mL^−1^) for 10 min. After 3 times rinses with PBS, the cells were observed and imaged by a fluorescence microscope.

### 2.7 *In vitro* cytotoxicity and apoptosis experiment

Cell counting kit-8 (CCK-8, Dojindo, Kyushu Island, Japan) assay was employed to evaluate the cytotoxicity *in vitro*. In brief, cells were seed at a density of 5,000 cells/well in 96-well plates and culture at 37°C for 24 h. Then, the cells were maintained in dark for another 24 h. After the corresponding treatment, the culture medium was then replaced with 100 μL of the corresponding culture medium containing 10% CCK-8 solution and cultured at 37°C for 2 h. Finally, the absorbance was measure at 450 nm using a microplate reader (Biotek, Winooski, VT, United States).

The apoptosis-inducing effect was determined by Annexin V-FITC/propidium iodide (PI) Apoptosis Kit (Nanjing Keygen Biotech, Nanjing, China). Briefly, the cells were seeded in a 6-well plate at a density of 1 × 10^5^ cells/well and cultured overnight. After the indicated treatment and subsequent 24 h of incubation, the cells were collected and rinsed twice with PBS. The Annexin V-fluorescein isothiocyanate (FITC)/PI kit was employed for staining of the above cells according to the manufacturer’s protocol. After another 20 min incubation in the dark, the cells were analyzed by flow cytometry (Becton Dickinson, Franklin Lakes, New Jersey, United States).

### 2.8 *In vitro* ROS detection

The intracellular ROS production was detected by a ROS assay kit (Beyotime Company, Shanghai, China). In brief, the MNNG/HOS cells were seeded in a six-well cell culture plate (1 × 10^5^/well) and incubated for 24 h. After the corresponding treatment, cells were washed twice with PBS, incubated with DCFH-DA reagent (10 µM) in medium without FBS at 37°C for 30 min, and then washed with PBS three times. After that, the fluorescence intensity of the cells was detected by a fluorescence microscope.

### 2.9 *In vitro* mitochondrial membrane potential (MMP) and intracellular adenosine triphosphate (ATP) detection

The MNNG/HOS cells were firstly seeded and cultured for 24 h in six-well cell culture plates. Following 6 h of corresponding treatment, the cells were treated by an NIR laser for 10 min.

For detection of the change of MMP, the 5,5′,6,6′-tetrachloro-1,1′,3,3′-tetraethylbenzimidazolocarbocyanine iodide (JC-1) detection Kit was employed. Briefly, the cells were incubated with JC-1 (5 μg mL^−1^) for 20 min, washed by PBS and then observed by fluorescence. For detection of the cellular ATP, the ATP determination kit (Beyotime, China) was used. In brief, the treated cells were harvested and thoroughly lysed with the lysis buffer. Afterwards, the supernatant was obtained by 5 min of centrifugation at 12,000 rpm under 4°C and the absorption was recorded. Finally, the ATP levels of the samples were determined according to the standard curve plotted based on the manufacturer’s protocol.

### 2.10 *In vivo* fluorescence imaging and photothermal imaging

All animal procedures were approved by the Institutional Animal Care and Use Committee (IACUC) at Tongji Medical College, Huazhong University of Science and Technology.

MNNG/HOS tumor-bearing mice were obtained by subcutaneous injection with 200 μL of MNNG/HOS cell suspension at the density of 1 × 10^7^ cells/mL. When tumors grew to about 150 mm^3^, the mice were administered GO-PEG-FA/ICG *via* tail vein injection. After administration, the mice were scanned at the predetermined time and the tumor imaging was obtained by IVIS small animal imaging system (PerkinElmer Inc., Waltham, United States).

### 2.11 *In Vivo* antitumor effect and biosafety

MNNG/HOS tumor-bearing mice were obtained as above mentioned. When the tumor reached about 50 mm^3^, the mice were randomly divided into four groups: 1) PBS + NIR; 2) free BD + NIR; 3) GO-PEG-FA/BD; and 4) GO-PEG-FA/BD + NIR (n = 3 per group). 100 μL PBS or free BD (10 mg kg^−1^) was injected intraperitoneally every 3 days. Other therapeutic agents were intravenously injected into the mice *via* the tail vein every 3 days. Six hours after injection, the tumor of all groups were irradiated with/without 808 nm laser for 5 min. The mice were weighted and measured with caliper every other day. Tumor volumes were calculated as length × width^2^/2 (mm^3^).

At the end of the treatment, the tumors were collected. Hematoxylin and eosin (H&E) staining assay and terminal deoxynucleotidyl transferase-mediated dUTP Nick-End Labeling (TUNEL) assay were performed to assess the anti-tumor efficacy of different groups. To evaluate the safety of all the groups, the major organs (heart, liver, spleen, lung, and kidney) were harvested at the end of treatment and stained with H&E staining. Furthermore, the plasma was also collect to perform blood chemistry analysis.

### 2.12 Statistical analysis

All of the experimental data were analyzed at least three independent experiments under the same experimental conditions. The results were expressed as the mean values ±standard deviation (SD). All the statistical analyses were tested by using Student’s t-test and one-way analysis of variance (ANOVA) conducted on the software GraphPad Prism version 6.01 for Windows. A *p*-value <0.05 was considered as statistically significant.

## 3 Results and discussion

### 3.1 Synthesis and characterization of NPs

**FIGURE 1:**
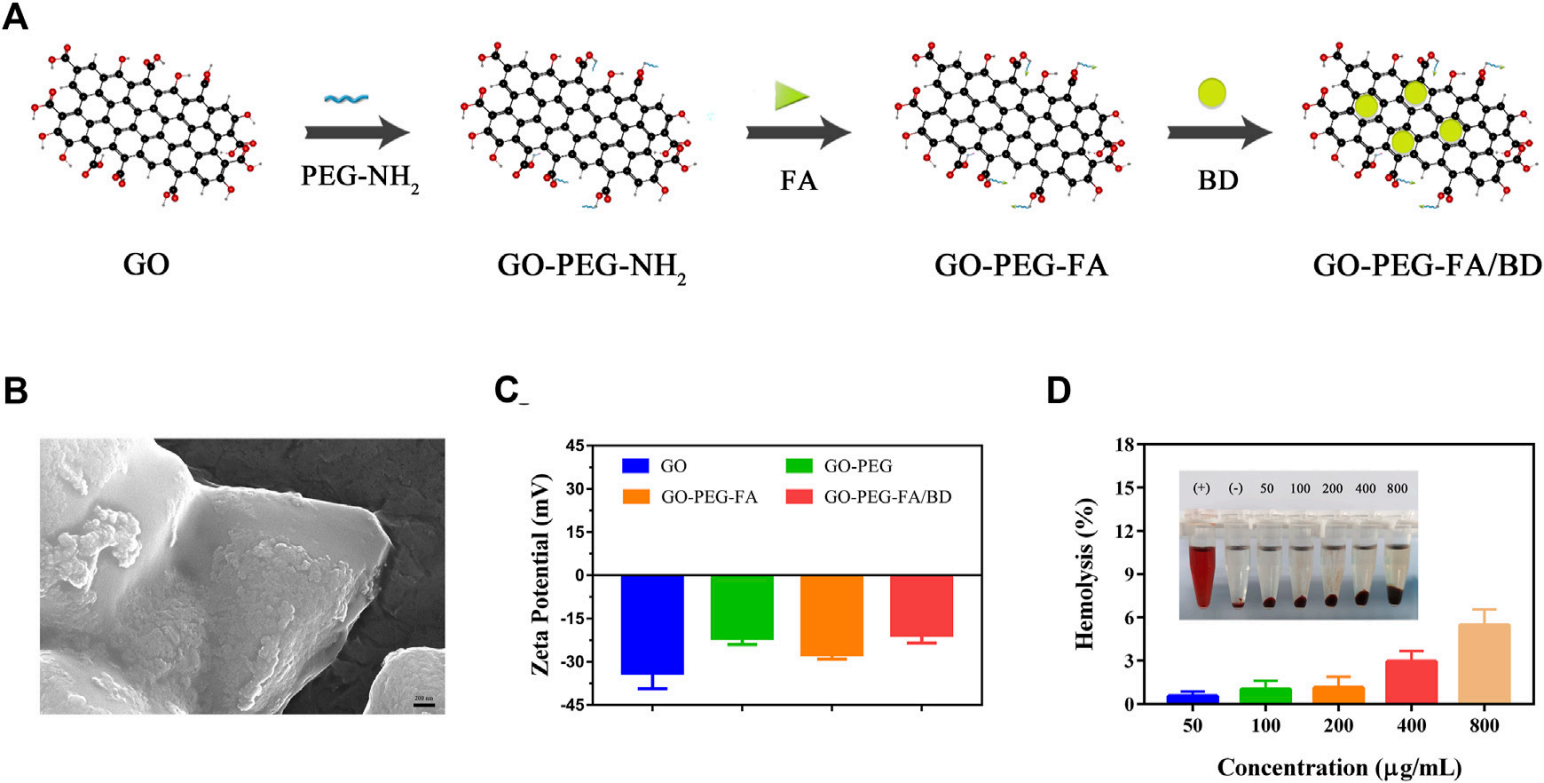
**(A)** Illustration of synthesis process of the nanoparticles. **(B)** SEM image of GO-PEG-FA/BD. **(C)** Zeta potential of GO, GO-PEG, GO-PEG-FA and GO-PEG-FA/BD. **(D)** Hemolysis analysis of the NPs.

The detailed design of GO-PEG-FA/BBRG was showed in Figure 1A. Firstly, GO was synthesized using the modified Hummers method. As shown in Figure 1B, the morphology of as-prepared GO-PEG-FA/BD was characterized by SEM. As demonstrated in Figure 1C, these altered zeta potentials provided evidence for the successful synthesis of the NPs.

### 3.2 Photothermal properties of NPs *in Vitro*

Due the good photothermal conversion efficiency, GO was usually used as a photothermal agent for PTT against cancer (Guo et al., 2022). In order to investigate the photothermal properties of GO-PEG-FA/BD *in vitro*, an infrared thermal imaging camera was used for real time measurement of the temperature changes of NPs after NIR irradiation (808 nm). As displayed in Supplementary Figure S1A, the temperature of GO-PEG-FA/BD NPs exhibited a promising concentration-, time-, and laser power intensity-dependent manner. However, it could be found the temperature of PBS rose slightly under the same conditions. In addition, the photothermal stability of the NPs upon the NIR irradiation was further evaluated. As shown in Supplementary Figure S1B, the temperature variation curves and peak shape were of no significant change after five cycles of irradiation with an 808 nm laser. Overall, all these results suggested that the nanocomposites exhibited superior photothermal conversion property and excellent photothermal stability.

### 3.3 Biocompatibility of the NPs

The blood hemolysis assay was first applied to investigate the *in vitro* hemocompatibility of GO-PEG-FA NPs. As demonstrated in Figure 1D, no significant hemolysis of RBCs was observed in the GO-PEG-FA NPs even at the highest concentration of 800 μg mL^−1^. In marked contrast, severe RBCs hemolysis happened to the positive control (pure water). These results demonstrated that the GO-PEG-FA NPs possessed an excellent hemocompatibility for peripheral blood circulation. In addition, the cytotoxicity of GO-PEG-FA NPs was further assessed by CCK8 assay. As displayed in Supplementary Figure S2, GO-PEG-FA NPs showed less toxicity to MNNG/HOS cells and normal human cells (BMSCs) after 24 h treatment.

All these findings suggested that the as-synthesized GO-PEG-FA/BD could be utilized as an extraordinarily biocompatible nanoplatform for drug delivery.

### 3.4 Cellular uptake and mitochondrial targeting effects

**FIGURE 2:**
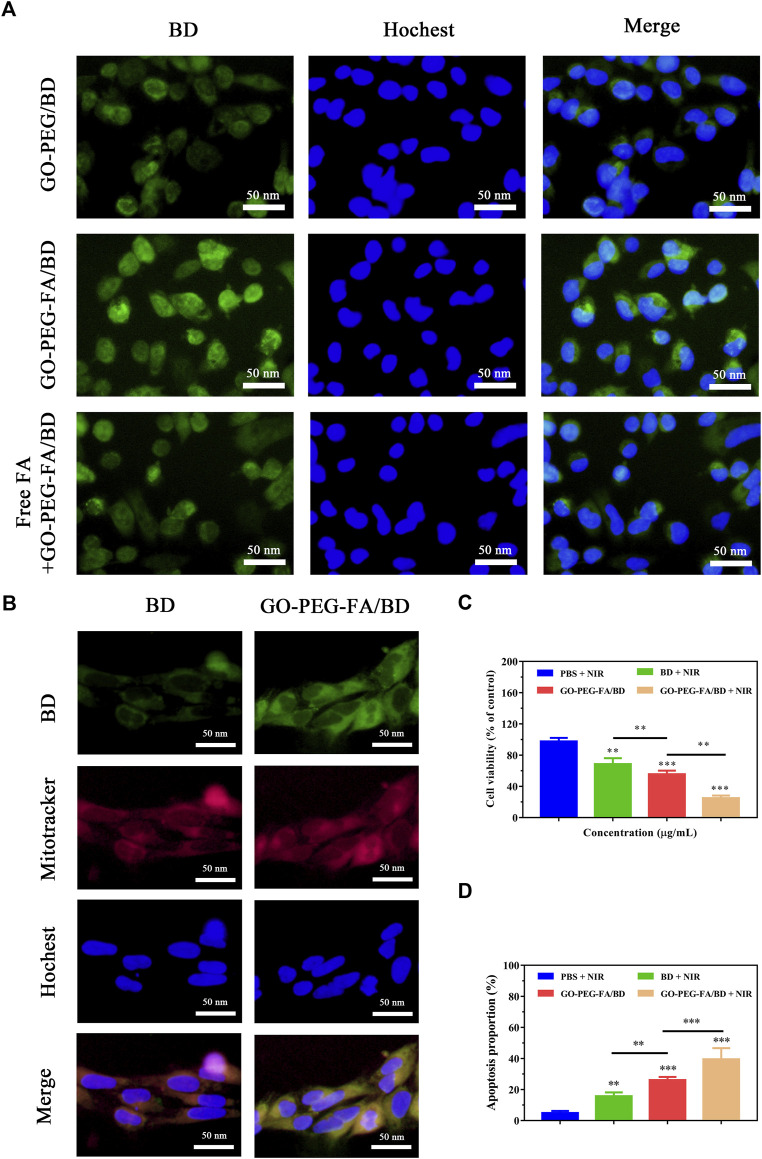
**(A)** Fluorescence images of MNNG/HOS cells after co-culture with various nanoparticles. **(B)** Co-localization of the NPs in the mitochondria of MNNG/HOS cells. **(C)** Cell viability after incubation with different concentrations of GO-PEG-FA/BD. **(D)** Quantified analysis of apoptosis after different treatments.

Numerous studies have shown that FA modified nanoparticles could target various cancer cells including OS (Hu et al., 2021; Mi et al., 2021). Accordingly, we modified FA molecules on the surface of the NPs (GO-PEG) hoping to achieve active tumor targeting by the specific interaction with FA receptor. In order to investigate the cellular uptake behavior of the NPs, cells were incubated with various drug formulations and then examined by using a fluorescence microscope. As shown in Figure 2A, the fluorescence intensity in MNNG/HOS cells treated with GO-PEG-FA/BD was dramatically stronger than that of the cells treated with GO-PEG/BD and free BD. In addition, the FA molecule competing assay illustrated that the uptake of GO-PEG-FA/BD was dramatically reduced by the pretreated free FA molecules. These results suggested that the enhanced uptake of GO-PEG-FA/BD might be partly mediated by FA-mediated endocytosis, which provided the potential for tumor-targeted drug delivery.

To further investigate the mitochondrial targeting effects of the nanocomposites, the sub-cellular localization of the NPs was evaluated by a fluorescence microscope. As shown in Figure 2B, free BD (emitted green fluorescence) could partially accumulate into the mitochondria stained as red fluorescence by Mito Tracker Red. Comparatively, higher green fluorescence in mitochondria was observed in cells treated with GO-PEG-FA/BD. This result further confirmed that the NPs exhibited the appreciable tumor target ability and excellent mitochondria-targeting ability.

### 3.5 *In vitro* synergistic efficacy

After demonstrating the excellent mitochondrial targeting ability of the NPs, the synergistic antitumor efficacy was first investigated *in vitro*. MNNG/HOS cells were treated with various formulations and determined with the CCK8 assay for cytotoxicity testing. As shown in Figure 2C, the formulation of GO-PEG-FA/BD showed higher cytotoxicity to MNNG/HOS cells than free BD under the same condition, which might be attributed to the enhanced internalization of nanodrugs through FA-receptor mediated endocytosis. Furthermore, GO-PEG-FA/BD couple with a low temperature laser irradiation displayed an enhanced cell-killing effect, indicating the nanocomposite with NIR laser irradiation achieved a prominent synergistic antitumor effect. Additionally, the apoptosis-inducing effect was quantitatively investigated by using flow cytometry with Annexin-V- FITC/PI double staining. As expect, GO-PEG-FA/BD under a low temperature laser irradiation could induce a dramatically higher level of cell apoptosis (Figure 2D). Collectively, all these results confirmed the prominent synergistic therapeutic effect of mitochondria-targeting LPTT.

### 3.6 Mitochondria damage and ROS enhancement

Mitochondria are vital metabolic organelles playing an essential role in regulating the adenosine triphosphate (ATP) production and intrinsic cell apoptosis (Sood et al., 2021). Therefore, these subcellular organelle is certainly to be considered as an excellent therapeutic target for cancer treatment. Increasing evidence has suggested that the mitochondria damage might directly activate the intrinsic mitochondrial apoptosis pathways, ultimately inducing cell apoptosis (Tan et al., 2018; Wang et al., 2019). It is hypothesized that mitochondria-targeting LPTT using GO-PEG-FA/BD could eliminate membrane potential (ΔΨm), deprive the ATP of cancer cells, and increase the levels of ROS, which ultimately induce cell apoptosis.

**FIGURE 3:**
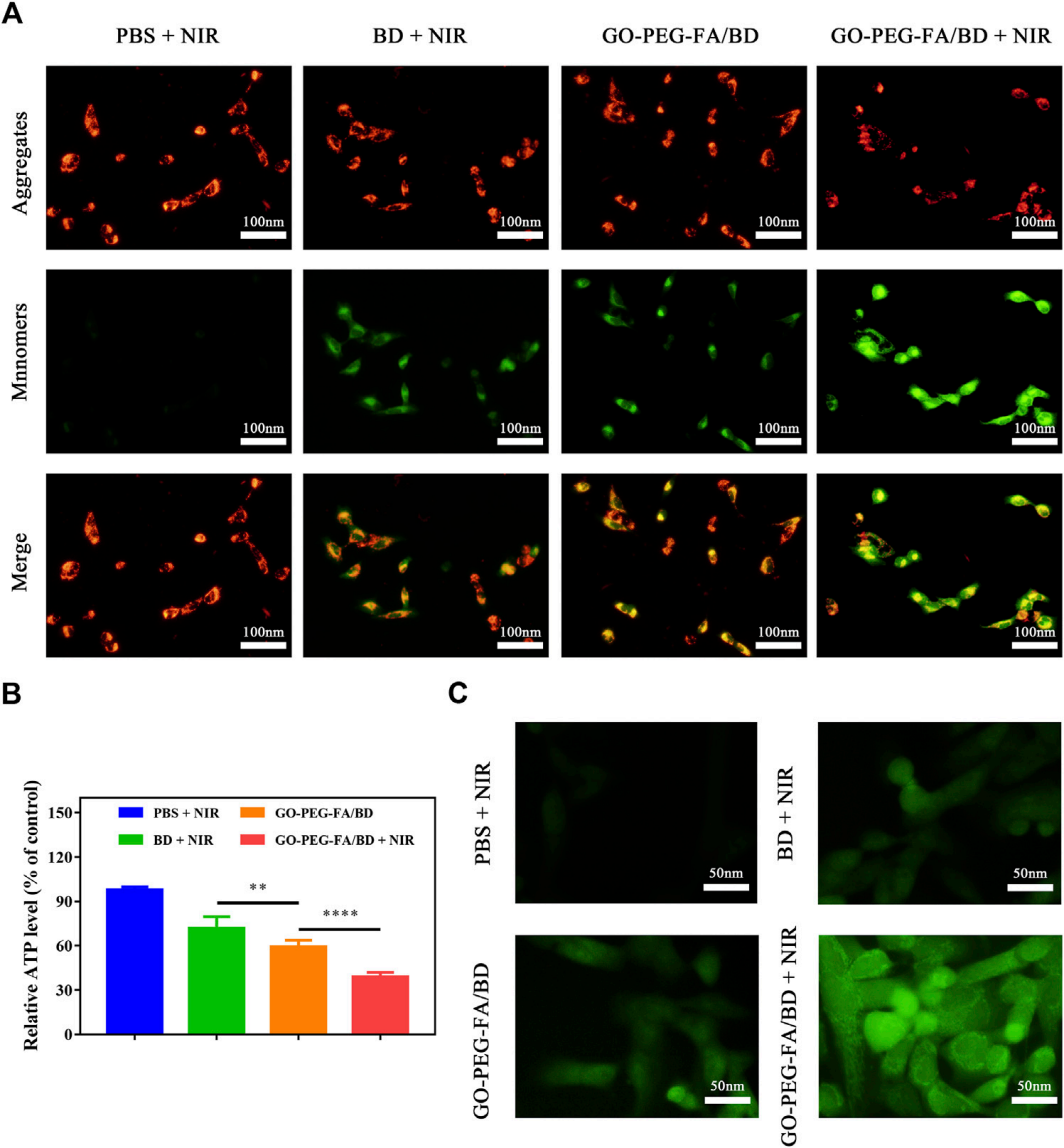
**(A)** Variation of the membrane potential of mitochondria after different treatments. **(B)** Relative ATP level of MNNG/HOS cells after corresponding treatments. **(C)** ROS generation of MNNG/HOS cells after various treatments.

The mitochondrial membrane potential changes were firstly investigated by using mitochondrial membrane potential assay kit with JC-1. As shown in Figure 3A, GO-PEG-FA/BD couple with a low temperature laser irradiation exhibited the strongest green fluorescence, indicating the destruction of membrane integrity. Meanwhile, ATP levels were significantly reduced in the cells treated with GO-PEG-FA/BD couple with a low temperature laser irradiation (Figure 3B). Consistent with these results, we found that GO-PEG-FA/BD + NIR showed the greatest potency in stimulating the production of ROS (Figure 3B). In brief, all the results above exhibited that MNNG/HOS cells treated with GO-PEG-FA/BD couple with a low temperature laser irradiation underwent the mitochondrial depolarization, ATP deprivation, and substantial ROS generation, ultimately inducing mitochondrial associated apoptosis in cancer cells.

### 3.7 *In vivo* distribution and photothermal effect

**FIGURE 4:**
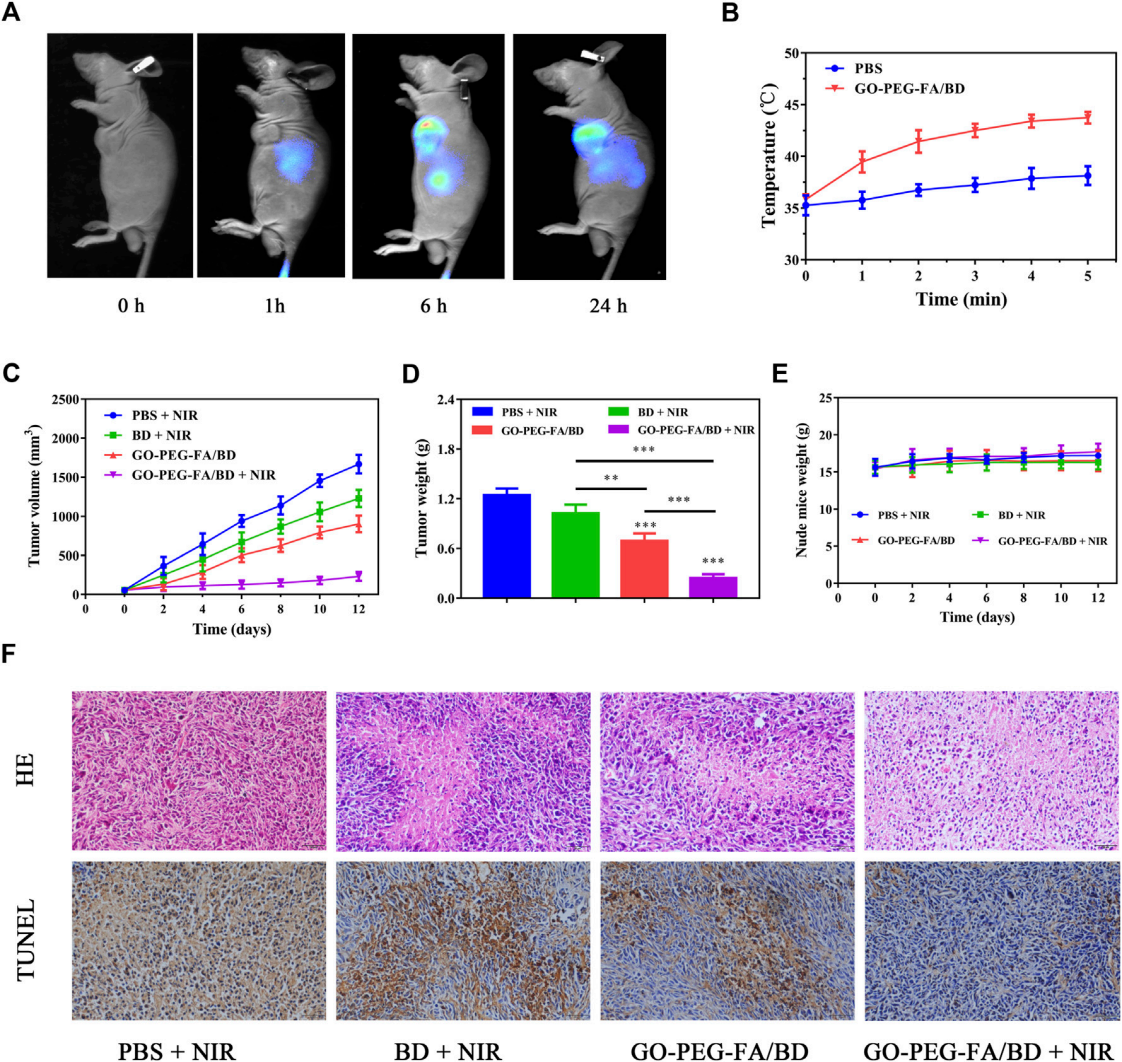
**(A)** *In vivo* fluorescence images of the tumor-bearing mice. **(B)** Temperature variation curves of the tumor region recorded by thermal camera during irradiation. **(C)** Tumor growth curves of the mice with different treatments. **(D)** Average tumor weights of the mice after corresponding treatments. **(E)** Body weight of the tumor-bearing mice during the treatment. **(F)** HE staining and TUNEL analysis of the tumor tissues from different groups.

To evaluate the *in vivo* distribution of the nanocomposites, we firstly established the MNNG/HOS tumor-bearing mouse model. Subsequently, the mice were intravenously injected with ICG labeled GO-PEG-FA (GO-PEG-FA/ICG) and then observed at the predetermined time by an IVIS small animal imaging system. As illustrated in Figure 4A, the fluorescence signals in the tumors gradually increased over time and the fluorescence intensity reached a maximum at 6 h post-injection. It was worth noting that the tumor site still remained the strongest fluorescence after 24 h administration, indicating the long retention of the NPs. The efficient tumor enrichment effect might be ascribed to the enhanced permeability and retention (EPR) effect-induced passive targeting and FA-mediated active targeting delivery (Park et al., 2019; Zhao et al., 2019; Hu et al., 2021).

Based on the above findings, we chose 6 h post-injection as the optimal time point for photothermal treatment. After 6 h post-injection, photothermal images from the tumor were taken to monitor the changes of temperature. As displayed Figure 4B, the local tumor temperature of the mice treated with GO-PEG-FA/BD could reach about 45°C after irradiation at 1.0 W cm^−2^ for 5 min. However, the temperature in the PBS treated group increased slightly under the same treatment conditions. These findings demonstrated that the nanocomposites could be employed to realize the quick increase of the temperature *in vivo*.

### 3.8 *In vivo* synergistic antitumor efficacy

**FIGURE 5:**
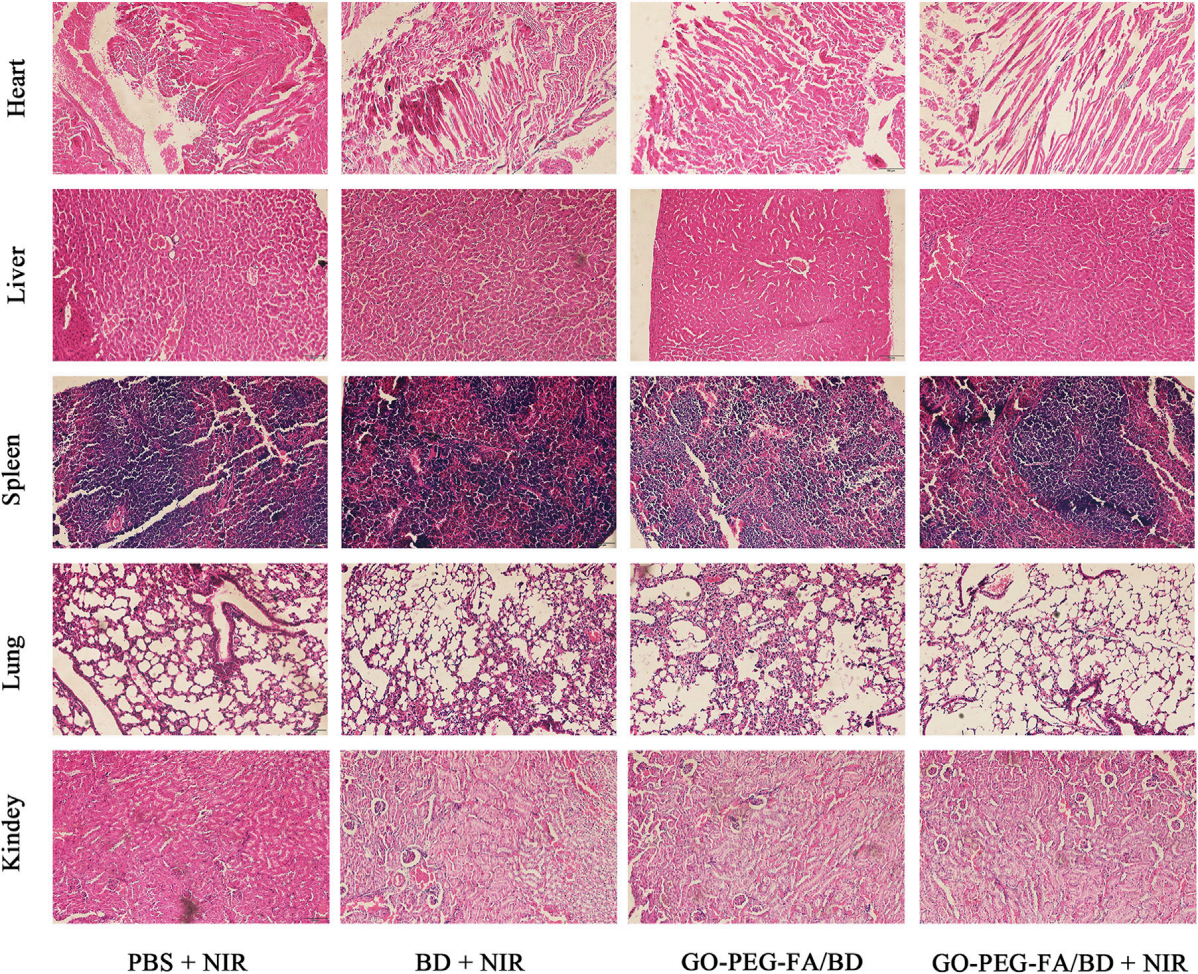
HE staining of major organs of the tumor-bearing mice after various treatments.

Encouraged by the effective tumor accumulation of GO-PEG-FA/BD and the satisfying anticancer effect *in vitro*, the *in vivo* synergistic combination antitumor therapeutic efficiency was further evaluated by a tumor-bearing mouse model established using MNNG/HOS cells. As indicated in Figures 4C, D, the mice treated with GO-PEG-FA/BD + 808 nm laser irradiation showed remarkable inhibition on tumor growth, which verified the outstanding synergistic therapeutic effect. Of note, there was no significant change in the weight of the mice in all groups during the 14 days of monitoring (Figure 4E). In addition, no significant tissue damage was observed in all the groups, confirming the favorable biological safety of all the treatments (Figure 5).

To further confirm the synergistic therapeutic efficacy *in vivo*, the tumors were harvested for HE staining and TUNEL assay (Figure 4F). In HE staining, the tumor tissue from the mice treated with GO-PEG-FA/BD + NIR showed the largest area of tumor necrosis compared to other groups, which was in good agreement with the anticancer results. As exhibited in the TUNEL staining images, the significant cell apoptosis was observed in tumor tissues treated with GO-PEG-FA/BD + NIR. Meanwhile, the liver and kidney function was evaluated by determining the serum level of alanine aminotransferase (ALT) and blood urea nitrogen (BUN), respectively. As shown in Supplementary Figure S3, the levels of ALT and BUN were all within normal range. Over all, all these results collectively certified the excellent synergistic antitumor effects and remarkable systemic safety of the mitochondria-targeting LPTT.

## 4 Conclusion

In summary, the current work provided a simple and facile nano-precipitation method by which a mitochondria targeted photothermal nanoplatform (GO-PEG-FA/BD) with high biocompatibility has been successfully developed. Benefiting from the active targeting capability of FA and mitochondria-targeted ability of BD, GO-PEG-FA/BD could selectively accumulate in mitochondria of cancer cells. As expected, GO-PEG-FA/BD couple with a low temperature laser irradiation exhibited an enhanced synergistic anticancer effect *in vitro* by activating the mitochondrial apoptosis pathway. Furthermore, *in vivo* studies also confirmed that the presented excellent *in vivo* antitumor ability without appreciable systemic toxicity. In conclusion, this study provides a practicable strategy to develop an ingenious nanoplatform for cancer synergetic therapy *via* mitochondria-targeting LPTT, which hold enormous potential for future clinical translation.

## Data Availability

The original contributions presented in the study are included in the article/Supplementary Material, further inquiries can be directed to the corresponding authors.
