# Supplementary figures and images for: Oxidative stress induced by berberine-based mitochondria-targeted low temperature photothermal therapy
Source: Front Chem. 2023 Feb 2;11:1114434. doi: 10.3389/fchem.2023.1114434 (PMC9932336; doi:10.3389/fchem.2023.1114434)

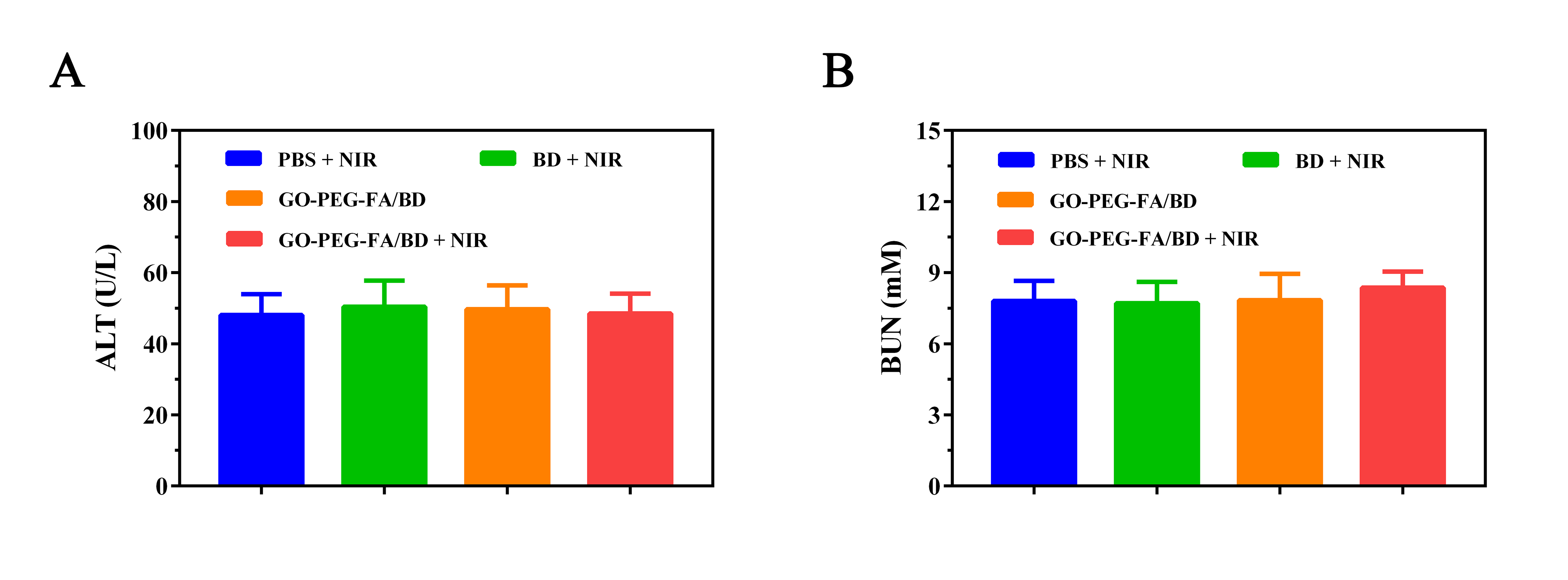

Supplement: Supplementary file 1 [file Image3.TIF]

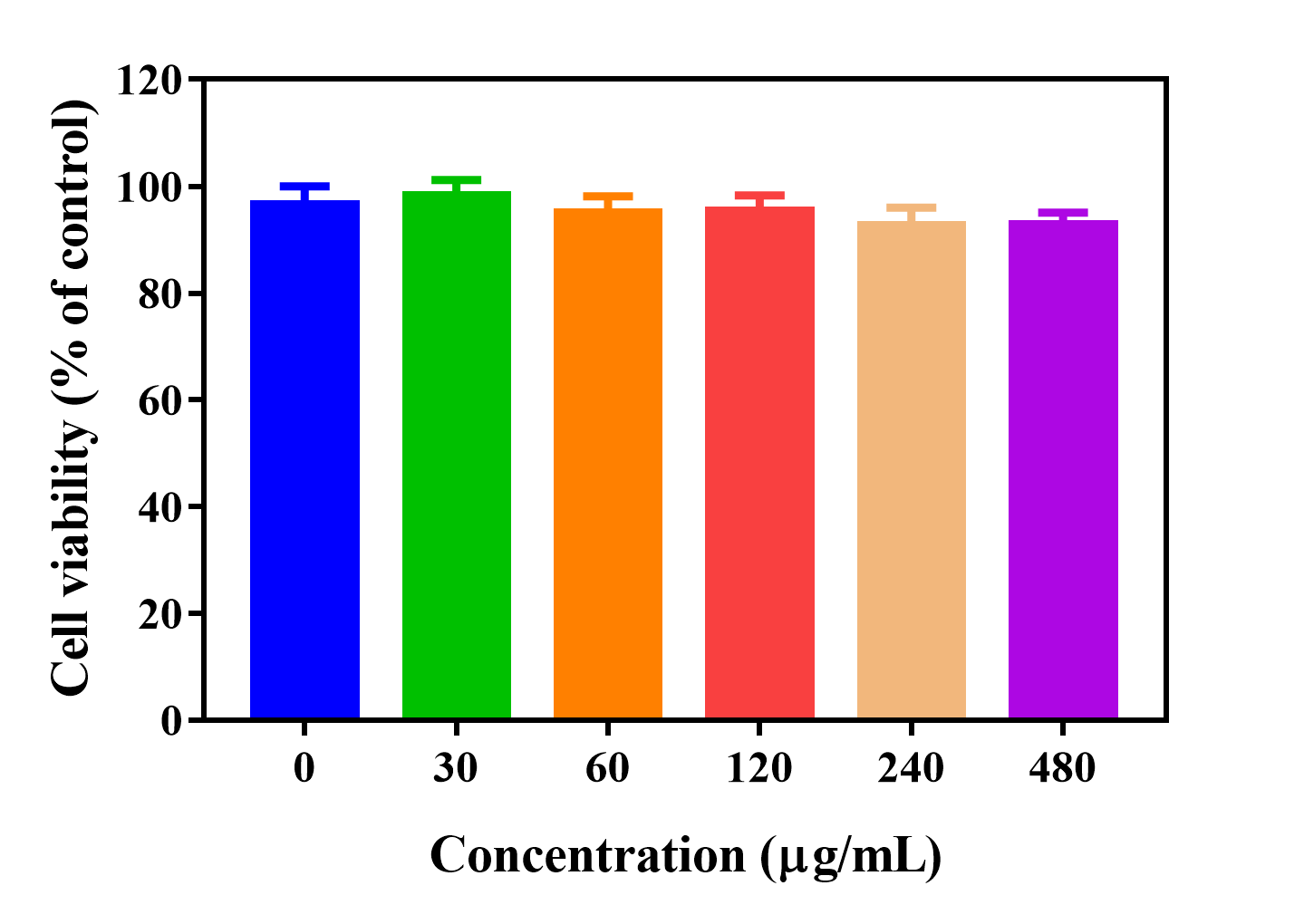

Supplement: Supplementary file 2 [file Image2.TIF]

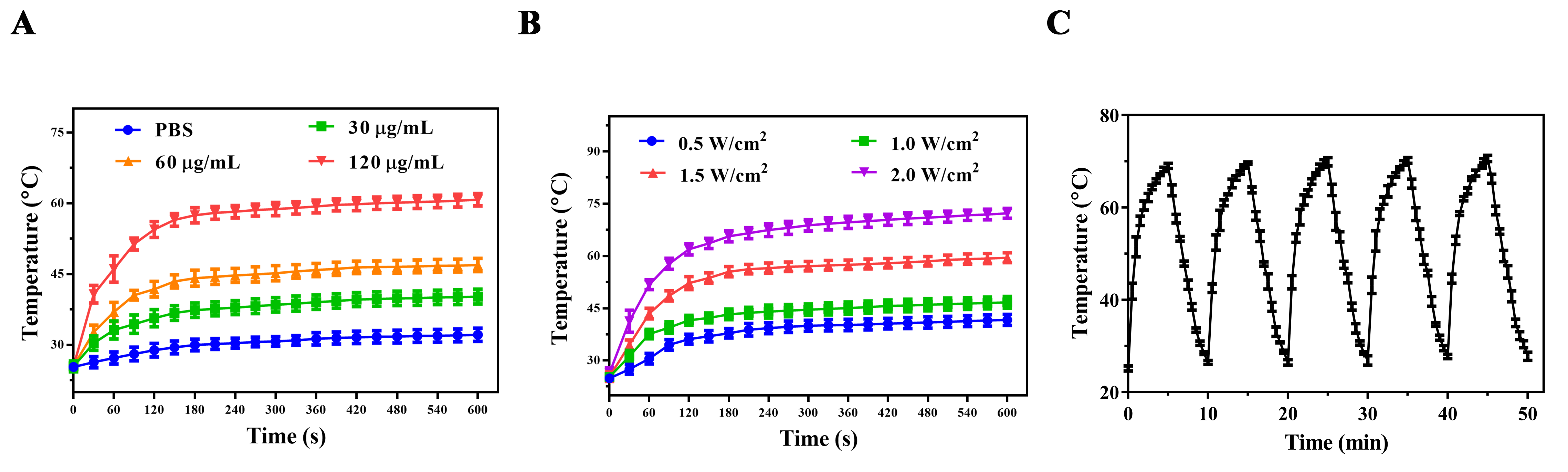

Supplement: Supplementary file 3 [file Image1.TIF]
